# Evaluating frailty scores across experimental groups in rodent models: bridging physical and cognitive domains

**DOI:** 10.1002/2211-5463.13955

**Published:** 2024-12-19

**Authors:** Aleksandra Mladenovic, Smilja Pracer

**Affiliations:** ^1^ Department for Neurobiology, Institute for Biological Research ‘Sinisa Stankovic’, National Institute of Republic of Serbia University of Belgrade Belgrade Serbia

**Keywords:** behavior, cognition, frailty, rodent

## Abstract

Frailty, a reversible clinical geriatric syndrome, impairs the ability to maintain homeostasis, leading to severe consequences such as hospitalization and death. Cognitive frailty, characterized by the co‐occurrence of physical frailty and cognitive impairment, has garnered increasing attention in recent years. Preclinical models, especially rodent studies, are essential for understanding frailty and developing interventions to mitigate associated conditions. Traditionally, animal studies have focused solely on physical frailty. We have pioneered the inclusion of cognitive parameters by developing a novel physical‐cognitive frailty score (FS) in animal research, in order to assess the effectiveness of anti‐aging interventions. Here, we provide a detailed example of the FS calculation at the group level, which can serve as a guide for other studies. This dual‐focus approach also helps in understanding how physical frailty and cognitive impairment interact to exacerbate adverse health outcomes and provides an opportunity to evaluate potential interventions that target both physical and cognitive dimensions of frailty more reliably.

## Graphical abstract

Method for Frailty Score (FS) calculation: (1) Combine all animals that will be compared in one group; (2) Calculate 20% cutoff; (3) Determine the number of animals that ‘failed’ or ‘passed’; (4) Calculate ‘percentage of success’ for graphical representation of parameters; (5) Repeat for all parameters; (6) Calculate FS and use percentages for graphical representation of FS.

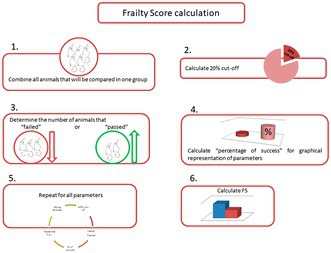

**Abbreviations**
AT: total duration of movement‐ambulatory time
CF: cognitive frailty
DR: dietary restriction
DT: total distance traveled
DT/AT: average velocity of movement
ER: exploration ratio
FI: frailty index
FS: frailty score
LTM: long‐term memory
NOR: novel object test
OF: open field test
SAB: spontaneous alterations
STM: short‐term memory
T/AT: percent of total time spent in moving
V1B: rearing frequency i.e. vertical activity

Frailty, included among the most serious public health challenges of our century [1], is broadly defined as a clinical geriatric syndrome, implicated in both poor quality of life, and negative health outcomes leading to loss of physiological reserves and serious consequences such as hospitalization, disability, and death [2] Since it was first reported in the geriatric field in the 1950s and 1960s, the frailty concept significantly evolved [3]. While the concept of physical frailty has been recognized as an important, valuable, and well‐defined entity for decades, the concept of cognitive frailty (CF) is gaining increasing attention and it has been used in geriatric in recent years. According to International Academy on Nutrition and Aging and the International Association of Gerontology and Geriatrics, CF includes both physical frailty and cognitive impairment [4, 5, 6]. However, the concept remains controversial. Although a significant effort was made to recognize cognitive frailty as a distinct syndrome integrating both physical frailty and cognitive impairment, some researcher still argue that cognitive frailty is merely a co‐occurrence of frailty and cognitive decline without sufficient evidence to justify it as a separate clinical entity [7]. This ongoing debate highlights the need for further research to clarify the interactions between physical and cognitive domains in the broader context of frailty.

In humans, the assessment of cognitive frailty includes comprehensive tools such as the Mini‐Mental State Examination and the Montreal Cognitive Assessment, and only recently, cognitive frailty screening tool was developed and used in a clinical study [8]. In the animal research, it is still not defined what kind of cognitive tools need to be included to better understand the interplay of frailty and cognitive decline [4, 9, 10, 11].

Experimental models are crucial in understanding frailty and developing effective interventions, ultimately guiding clinical research and improving health outcomes in the aging population. These models allow researchers to study the biological mechanisms underlying frailty in a controlled environment, facilitating the identification of potential therapeutic targets. Animal models, particularly rodents, are excellent for mimicking human frailty and allow the study of genetic, molecular, and environmental factors that contribute to this syndrome. Therefore, it is crucial to establish reliable and valid measures of frailty in animal studies in order to translate the results to human populations.

There are two major tools for frailty assessment in humans. The phenotype Frailty Score (FS), developed by Fried *et al*., includes five criteria: unintentional weight loss, slow walking speed, self‐reported exhaustion, weakness, and low physical activity [12]. In contrast, the Frailty Index (FI) defined by Rockwood and colleagues takes into account dozen of health deficits, including nonphysical ones [13]. Both tools for measuring human frailty have been transferred to animals, with a focus on assessing physical parameters. The first preclinical studies of frailty were conducted in mice and include both phenotypic (FS) and clinical approach (FI) [14, 15, 16, 17]. This was followed by the work of Miller and colleagues [18] and Yorke *et al*. [19] who adapted the frailty phenotype assessment and the clinical frailty index for use in rats. These developments opened the door to refining the frailty assessment, allowing it to be tailored not only to various species but also to the specific experimental paradigm and/or method for evaluation. Although frailty index tools encompass a diverse range of health deficits—many of which are not traditionally classified as physical—preclinical frailty assessments focus primarily on physical frailty. Notably, none of these instruments incorporate cognitive parameters. At the same time, clinical studies suggest that cognitive frailty is a reliable predictor of falls, injury, and disability, more than a physical frailty alone [20]. Given the reasons outlined above, we have developed a unique physical‐cognitive frailty score that incorporates both cognitive and physical parameters, reflecting the frailty levels at the group level in experimental populations. Using a battery of behavioral tests for evaluating both motor and cognitive performances of laboratory rodents, we were able to test the outcomes of various interventions against cognitive and physical decline.

In the following text, we use two of our previous studies as examples and provide a detailed description of the behavioral tests and parameters that can be incorporated into the frailty score [9, 11], along with the methodology for their calculation, using hypothetical data for illustration.

## Materials

### Animals

All animal handling has been described previously in detail, including all necessary documents and approvals [9, 11]. Briefly, male and female Wistar rats of different age (6, 18, and 24 months old) were used in these studies. All animal procedures complied with the EEC Directive (2–59/12) on the protection of animals used for experimental and other scientific purposes and were approved by the Ethical Committee for the Use of Laboratory Animals of the Institute for Biological Research ‘Sinisa Stankovic’, University of Belgrade and by the National Ethics Research Committee (#323‐07‐03065/2020–05, #323‐07‐13 536/2020‐05).

### Methods

Behavioral testing included open field test (OF), novel object test (NOR), and Y‐maze test [9, 11].

#### Open field test

Rats were allowed to freely explore the test arena for 10 min. For the calculation of frailty, we included 5 parameters from OF (total distance traveled, DT; total duration of movement‐ambulatory time, AT; percent of total time spent in moving, T/AT; average velocity of movement, DT/AT; rearing frequency i.e. vertical activity, V1B).

#### Novel object recognition task (NOR)

**Fig. 1:**
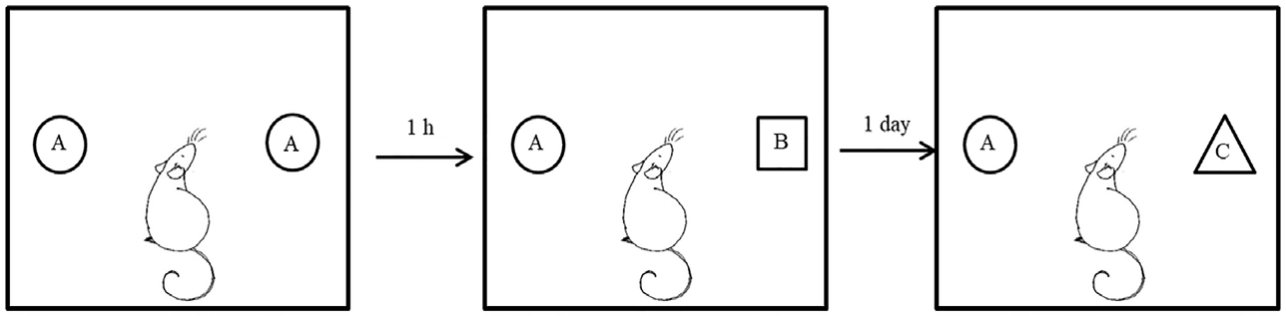
Graphical representation of Novel object test.

Novel object discrimination test (Fig. 1) was used for short‐term (STM) and long‐term memory (LTM) assessment. The day after the OF test, when animals had a chance to habituate to an empty test arena of the OF for 10 min, their memory performance was tested. Experimental procedure included four sessions:

- Pretrial habituation: animals were left to freely explore the empty OF arena for 10 min on the testing day;
- Familiarization trial: two identical objects (A + A) were placed in opposite corners of the arena to be explored by animals for 10 min;
- Choice trial 1: After 1 h, animals were exposed to STM testing for 10 min, where one object from familiarization trial was replaced by a novel one while the other was left untouched (A + B);
- Choice trial 2: After 24 h, animals were exposed to LTM testing for 10 min, where one object from familiarization trial was replaced by a novel one while the other was left untouched (A + C).

Exploration ratio (ER) was calculated regarding time spent exploring new versus familiar object, using the following formulas: ER = *t* novel/(*t* novel + *t* familiar) [24].

#### Y‐maze test

**Fig. 2:**
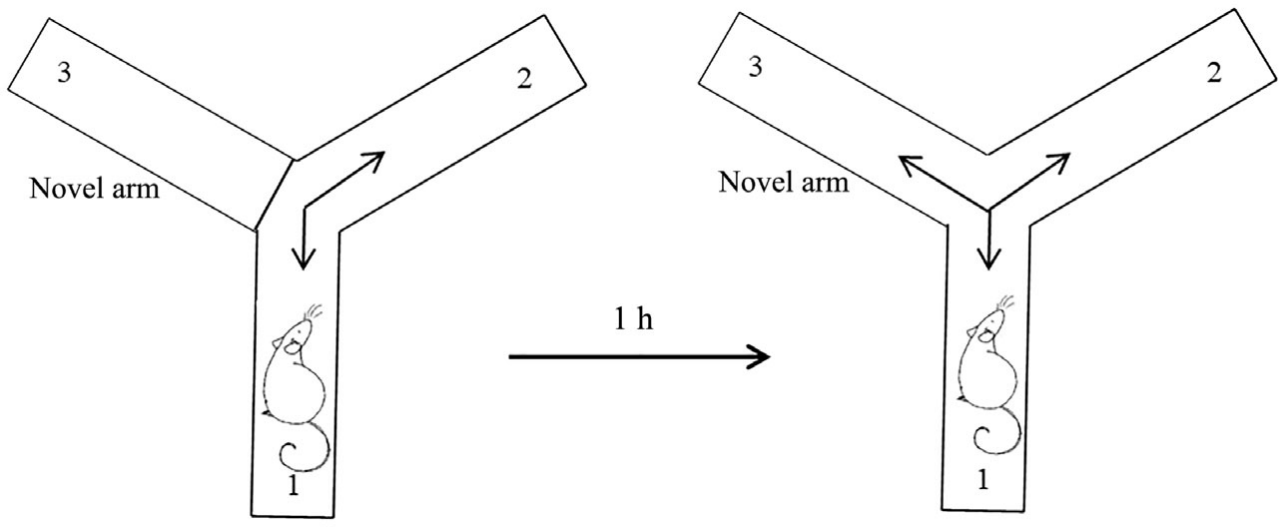
Graphical representation of Y‐maze test.

Animals were allowed to freely explore the two arms of Y‐maze (Fig. 2) during a 10‐min session, without any reinforcements, while the 3rd arm (novel arm) was blocked off during this first trial. After an intertrial interval of 1 h, rats were allowed to freely explore all three arms of the maze for the next 10 min (second trial). Total number of entries, time spent in a novel arm, number of entries in the novel arm, and % of novel arm entries were monitored and spontaneous alterations (SAB) from the second trial were calculated.

#### Frailty score, FS

We calculated the frailty score based on the ‘Valencia score’ developed by Gomez‐Cabrera *et al*. [17], who adapted Fried's score for humans [12] to experimental animals and which measures frailty at the group level. We further modified it by adding a cognitive component [9, 11]. We used five parameters from the open field test, two NOR parameters and five Y‐maze parameters (Table 1).

**Table 1:** Parameters used for physical‐cognitive FS calculation.

Test used for FS calculation	Type of parameter from different tests used for FS calculation	
	Cognitive	Physical
Open filed test	Spontaneous exploratory behavior as a measure of preserved cognition (Vertical activity, V1B)	Total activity (Distance traveled, DT) Ambulatory time (total duration of movement), AT Percent of total time spent moving, T/AT Average velocity of movement, DT/AT
Y‐maze	Short‐term memory (Number of entries in the novel arm) Time spent in the novel arm % of novel arm entries (Spontaneous alterations, SAB)	Total activity (Total number of entries)
Novel object recognition	Exploration ratio (ER) Short‐term memory Exploration ratio (ER) Long‐term memory	

**Fig. 3:**
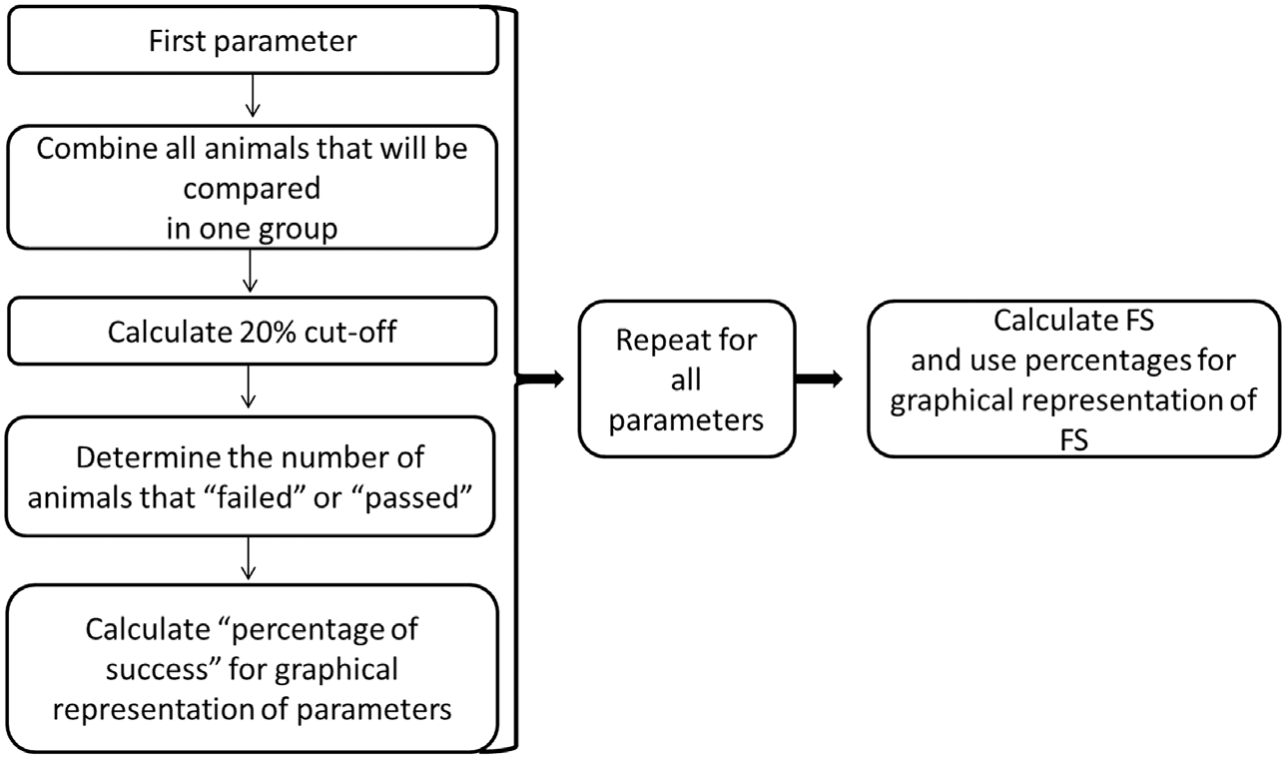
Schematic representation of the steps for group FS calculation.

When we define what parameters that will be used for group FS calculation, we determine the FS for each experimental group instead of determining FS for each animal. First, all animals from experimental groups that we want to compare (two or more) are combined into one group for statistical analysis. Using descriptive statistics, a cutoff value of 20% is determined for each parameter included in frailty score [17, 21, 22]. All animals for which the value of the respective parameter is below the cutoff value are marked as ‘failed’. Animals whose value were equal to or higher than the cutoff value are marked as ‘passed’ for the respective parameter. Next, we need to determine the ‘percentage of success’ for each parameter (Table 2) using the formula: Number of animals passed/(number of animals passed + number of animals failed) * 100. The number of failed animals in each experimental group for each parameter is then determined. Further, the FS for each experimental group was calculated as follows: Total number of animals that failed all the tests in each experimental group (A) divided by the total number of ‘passed’ animals (B) and expressed as a percentage (Fig. 3).

**Table 2:** Calculation of number of frail animals in each frailty group for DT parameter.

Parameter	Distance traveled, DT (cm)		
Experimental groups	Young	Middle‐aged	Old
Animal 1	390	479	593
Animal 2	**227**	**178**	1272
Animal 3	755	903	**347**
Animal 4	679	413	1369
Animal 5	501	904	**322**
Animal 6	1040	**129**	914
Animal 7	685	483	1757
Animal 8	1165	575	**307**
Animal 9	1304	780	**366**
Animal 10	729	**303**	719
Animal 11	852	900	809
Number of animals	11	11	11
20% Percentile cutoff value	**370.8**		
Number of animals failed **(A)**	1	3	4
Number of animals passed **(B)**	10	8	7
Percentage of success	91	73	64

Cut‐off method used in this step doesn not include p‐values.

Tables 2 and 3 provide an example using hypothetical data to illustrate the process of calculating the frailty score in detail. If we want to compare the FS between different age groups, young (6‐month‐old), middle‐aged (18‐month‐old) and old (24‐month‐old) animals, group all animals together and calculate the cutoff value for each parameter (DT, V1B… etc.). Let us start with the parameter DT (Table 2). DT value for each animal is determined and presented in the respective column (young, middle‐aged, and old). Calculation of cutoff value of 20% for all three age groups gives a value of 370.8 cm (Table 2). Comparison of a DT value of individual animals with this cutoff value clearly marks each animal as failed (DT value is below 370.8) or passed (DT value equal or higher than 370.9). In this manner, we determine how many failed and passed animals are in each age group (‘number of animals failed, (A)’, Table 2, bold values: young: 1, middle‐aged: 3, old: 4 animals; ‘number of animals passed, (B)’, Table 2, young: 10, middle‐aged: 8, old: 7 animals). Finally, we calculate percentage of success (Table 2: young: 91, middle‐aged: 73, old: 64). The whole process is repeated for all physical and cognitive parameters used for the FS calculation, and therefore, cognitive parameters were calculated in the same manner as explained on the example of DT (Table 1, 5 parameters from the OF, 2 NOR parameters and 5 Y‐maze parameters).

**Table 3:** Calculation of FS.

Number of failed animals for each parameter	Young	Middle‐aged	Old
Distance traveled, DT (cm)	1	3	4
Vertical activity, V1B (beams)	0	5	7
Velocity DT/AT (cm/s)	1	4	4
Ambulatory time AT (s)	0	1	3
Exploration ratio (ER), long‐term memory	0	2	3
Exploration ratio (ER), short‐term memory	0	1	4
Total time spent moving AT/T (%)	2	4	2
Weight (g)	1	3	2
Spontaneous alterations, SAB	1	2	5
Total number of entries	2	2	5
Number of novel arm entries	0	2	4
Time spent in novel arm (s)	2	2	5
Novel arm entries/total arm entries (%)	1	3	4
Total number of failed tests (A)	12	39	58
Total number of performed tests (B)	86	74	67
A/B	0.14	0.53	0.87
Frailty score: A/B*100	14	53	87

After determining the individual animals that failed/passed test for the first examined parameter in every experimental group (Table 2), we sum up the number of animals that failed/passed that parameter in each experimental group (Table 3). This step is then repeated for every parameter, as shown in Table 3. After that, we calculate the number (summing) of all failed/passed animals for all tests in each experimental group (Table 3A and B). Then, we finally can determine the group FS, by dividing the ‘total number of failed tests for all parameters’ (A) by the ‘total number of performed tests for all parameters’ (B) for each experimental group and multiplying the resulting number by 100 to express it as a percentage (Table 3: young: 14, middle age: 53, old: 87).

### Statistical analysis

Analysis of group FS was performed by Pearson's chi‐squared test, whereas data for each of the parameters were performed using G‐test with William's correction for small number of samples. It should be noted that errors cannot be presented on graph bars, due to the use of Pearson's chi‐squared test and G‐test. Statistical analysis was performed by using GraphPad Software (San Diego, CA). *P*‐value differences were considered to be statistically significant when *P* < 0.05.

## Results and discussion

- Graphical representation of each of the parameters used for group FS calculation presented as % of success (% of animals that performed test, e.g., did not fail that test). Usually, these graphs are given in the supplementary material.
- Main result: Graphical representation of the FS for each group that was evaluated.

The details of the statistical analysis and the presentation of the data are explained in the example below:

### Statistical analysis and graphical representation of parameters

Step 1: For the G‐test analysis, arrange the values in the table as follows: In the first column, enter in one row the number of animals in the group that failed the test, and in the next row, the number of animals that performed the test in the same experimental group, for example, ‘Young’ animals. In the second column (on the right), enter the values for failed/passed tests in the second experimental group, for example, ‘Middle‐aged’ and calculate the *P*‐value.

Step 2: Repeat the process for all comparisons for each parameter used.

**Fig. 4:**
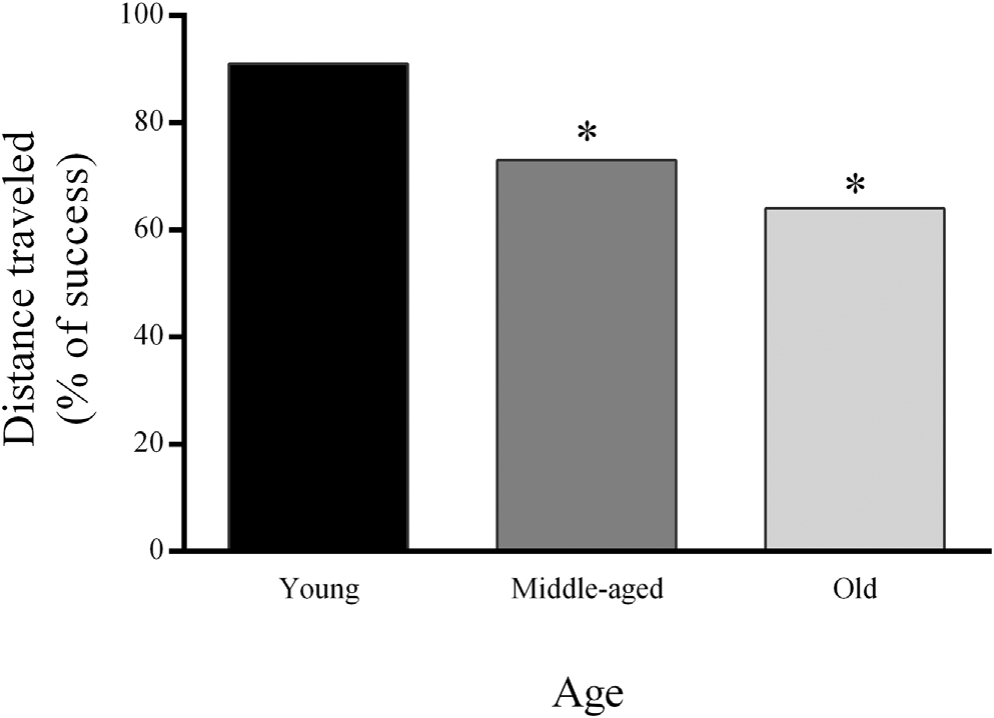
Example of graphical representation of OF parameter (DT) used for determination of frailty score (FS) during aging in young, middle‐aged, and old animals at group level. Data are expressed as percentage of animals that successfully passed the test. Statistical differences were tested using G‐test with William's correction. * represents statistical significance in comparison to young animals, *P* < 0.05.

For the graphical representation, calculate the percentage of success but use the percentages as values to create a graph (Fig. 4).

### Statistical analysis and graphical representation of FS

Arrange the values for Pearson's chi‐squared test in the table in the same way as explained for the G‐test (Table 4).

**Table 4:** Data organization for analysis of parameters/FS by G‐test/Pearson's chi‐squared test.

	Young	Middle‐aged
Failed	8	39
Passed	86	74
	Young	Old
Failed	8	58
Passed	86	64
	Middle‐aged	Old
Failed	39	58
Passed	74	67

**Fig. 5:**
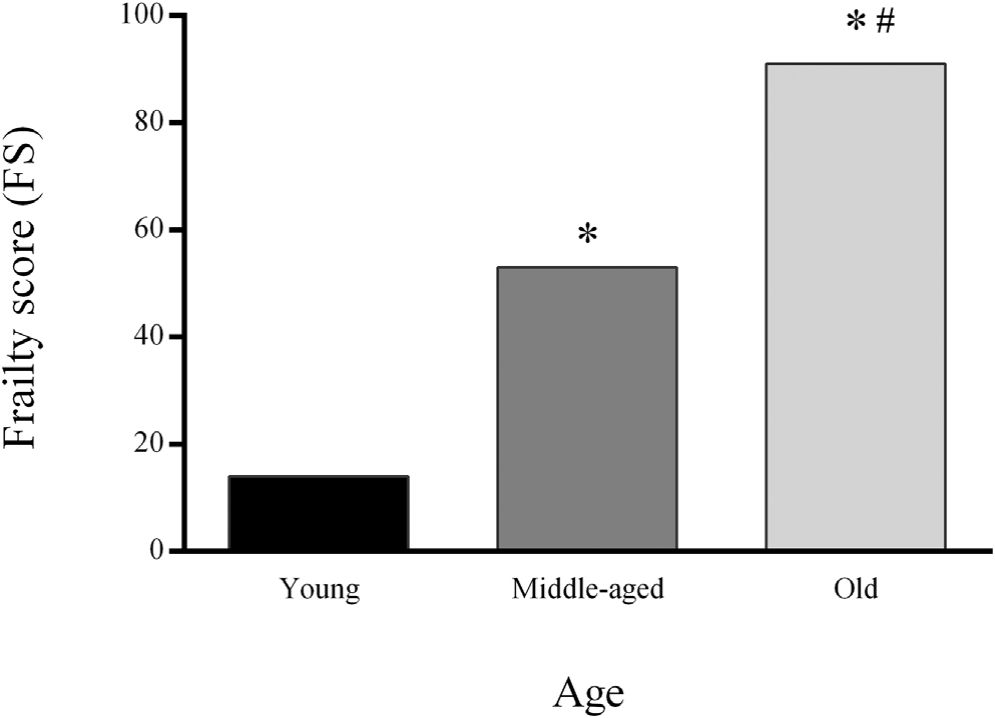
Example of graphical representation of group FS during aging in young, middle‐aged and old animals. Statistical differences were tested using Pearson's chi‐squared test. * represents statistical significance in comparison to young animals; # represents statistical significance in comparison to middle‐aged animals, *P* < 0.05.

Use the FS percentages as values for the graphical representation of the group FS (Fig. 5).

## Tips and tricks

### Battery of behavioral tests for FS assessment

It is not necessary to use the same tests in order to measure FS, and this is one of the strengths of using frailty score as a tool [23]. Different types of cognitive and motor tests can be used (individually and in combination). For example, in the paper Prvulovic *et al*. [11] we used OF for measuring physical aspect of FS, and combination of OF, NOR, and Y‐maze test for cognitive aspect of FS. In the study of Todorovic *et al*. [9] we used OF parameters for physical aspects of FS and OF and Y‐maze test for cognitive aspect of FS. Each test has its limitations and advantages, so the choice of the tests is not by chance, but it should be tailored so it can detect even subtle strain‐specific and age‐specific behavioral changes in different rodent strains [24].

An interesting concept of ‘mild frailty’ has been introduced by Liu *et al*. [16] that corresponds to prefrail state in humans. Implementing a prefrail stadium in the phenotypic frailty assessment would greatly improve sensitivity in animals and would enable identification of the animals whose values are near the cutoff. This approach would align with the Fried phenotypic frailty model [12], which already includes a prefrail state to capture subtle physiological declines before full frailty onset.

### Inclusion of body weight in FS assessment

Unintentional weight loss is one of the hallmarks of human frailty and is used as a parameter in FS assessment. If the experimental treatment affects one of the parameters, this parameter should be carefully addressed. For example, in the article by Todorovic *et al*. [9], we included body weight corrected for the effects of diet, but in the work with the female animals [11], we excluded this parameter due to the variation in the animals' body weight that could not be attributed to DR. The reason for this discrepancy could be that male and female rats have metabolic, physiological, and behavioral differences due to the influence of sex hormones affecting different body systems [25]. Several authors have utilized different cutoff values for body weight [16, 20, 21] because this parameter—and its corresponding thresholds—can be sensitive to various factors, including unintentional weight loss, obesity, age, and the species and strain of the animal. It is very important to consider the acceptability of each parameter used for FS calculation depending on the study protocol/treatment.

It is worth noting that in addition to using various behavioral tests to assess cognitive frailty, attempts have also been made to establish a correlation between cognitive impairment and various blood parameters in humans [26]. Furthermore, there is evidence that the progression of cognitive impairment in normal aging rats is associated with the increase in the level of DNA damage [27] which opens up the possibility of including other parameters in frailty research in order to achieve the most comprehensive assessment of frailty.

### Individual vs group FS

One limitation of the FS approach we used is that it measures the frailty of a group rather than individuals. Since age alone does not necessarily associate with frailty [28], not all animals of the same age may be frail or exhibit the same level of frailty. Individual frailty scores are particularly useful in longitudinal studies, where each animal serves as its own control and its frailty status is tracked over time, allowing for the detection of clear changes in frailty (e.g., from nonfrail to frail or vice versa). However, even in such studies, a group frailty score should be calculated at the end to assess how many animals in each group have become ‘frail’ over time.

On the other hand, group frailty scores are highly valuable in evaluating the effectiveness of interventions, as demonstrated in the studies we describe here. This approach was initially developed by Gomez‐Cabrera *et al*. [17], based on mouse and human frailty phenotype assessments, and calculates a frailty score for each age group of mice rather than for each individual mouse. Despite its limitations, this method represents a milestone in animal frailty research and makes a significant contribution to the field of animal frailty assessment [23], a field that we continue to develop by incorporating a cognitive component, as described in this manuscript.

### Frailty score versus frailty index

Researchers often face a choice between the two most commonly used tools for quantifying frailty: the frailty index approach and the frailty phenotype approach. We strongly recommend caution when selecting a frailty assessment tool. The frailty index measures the accumulation of health‐related deficits over an animal's lifetime, while the frailty phenotype assesses frailty based on performance in five functional criteria: weight loss, exhaustion, weakness, slowness, and lack of activity, evaluating how many of these criteria the animal performs poorly on [14, 15]. Both tools represent significant advances in gerontology, enabling preclinical investigations of anti‐aging and other interventions [29, 30]. Nevertheless, when choosing a frailty assessment method for a given experimental paradigm, we advise researchers to consider which approach aligns best with their objectives. In our previous work on dietary restriction, for example, we expected dietary restriction to primarily affect the physical characteristics of the animals rather than specific deficits in body systems, such as fur condition or eyesight. In these cases, group frailty score was the optimal approach. However, when possible, using both tools in tandem is preferable [31]. Since the frailty index and frailty phenotype measure different age‐related deficits, they may not always identify the same individuals or groups as frail. We demonstrated this in our previous study comparing these two tools in the 5xFAD transgenic mouse model, which shown as rather heterogeneous on several levels. Although we observed a significant increase in frailty with age in both sexes, the frailty index and frailty phenotype identified different mice as frail. This highlights the need for further refinement and adaptation of frailty measures in rodent models, especially in transgenic animals that mimic human diseases.

## Conflict of interest

The authors declare no conflict of interest.

## Author contributions

ST acquired the data, analyzed, and interpreted the data, AM and ST designed the project and wrote the paper.

## Data Availability

This manuscript presents a methodological protocol that describes the steps of the procedure without utilizing or relying on real or numerical data. The protocol itself is based on methods detailed in our previous publication, but no actual data were used in this work.
